# Prevalence, Motivations, and Social, Mental Health and Health Consequences of Cyberbullying Among School-Aged Children and Youth: Protocol of a Longitudinal and Multi-Perspective Mixed Method Study

**DOI:** 10.2196/resprot.5292

**Published:** 2016-05-24

**Authors:** Faye Mishna, Lauren B McInroy, Ashley Lacombe-Duncan, Payal Bhole, Melissa Van Wert, Kaitlin Schwan, Arija Birze, Joanne Daciuk, Tanya Beran, Wendy Craig, Debra J Pepler, Judith Wiener, Mona Khoury-Kassabri, David Johnston

**Affiliations:** ^1^ University of Toronto Factor-Inwentash Faculty of Social Work Toronto, ON Canada; ^2^ University of Toronto Dalla Lana School of Public Health Toronto, ON Canada; ^3^ Queens University Department of Psychology Kingston, ON Canada; ^4^ York University Department of Psychology, Faculty of Health Canada; ^5^ University of Toronto Ontario Institute for Studies in Education Toronto, ON Canada; ^6^ The Hebrew University of Jerusalem Mount Scopus School of Social Work & Social Welfare Jerusalem Israel; ^7^ Seneca College Toronto, ON Canada; ^8^ University of Calgary Cumming School of Medicine Calgary, AB, Canada

**Keywords:** cyberbullying, ICT, children, youth, parents, teachers, victimization, mental health, longitudinal, mixed methods

## Abstract

**Background:**

While the online environment may promote important developmental and social benefits, it also enables the serious and rapidly growing issue of cyberbullying. Cyberbullying constitutes an increasing public health problem – victimized children and youth experience a range of health and mental health concerns, including emotional and psychosomatic problems, maladaptive behaviors, and increased suicidality. Perpetrators demonstrate a lack of empathy, and may also struggle with health and mental health issues.

**Objective:**

This paper describes the protocols applied in a longitudinal and multi-perspective mixed-methods study with five objectives: (1) to explore children/youth’s experiences, and children/youth’s, parents’, and teachers’ conceptions, definitions, and understanding of cyberbullying; (2) to explore how children/youth view the underlying motivations for cyberbullying; (3) to document the shifting prevalence rates of cyberbullying victimization, witnessing, and perpetration; (4) to identify risk and protective factors for cyberbullying involvement; and (5) to explore social, mental health, and health consequences of cyberbullying.

**Methods:**

Quantitative survey data were collected over three years (2012-2014) from a stratified random baseline sample of fourth (n=160), seventh (n=243), and tenth (n=267) grade children/youth, their parents (n=246), and their teachers (n=103). Quantitative data were collected from students and teachers during in-person school visits, and from parents via mail-in surveys. Student, parent, and teacher surveys included questions regarding: student experiences with bullying/cyberbullying; student health, mental health, and social and behavioral issues; socio-demographics; and information and communication technology use. In-depth semi-structured qualitative interviews were conducted twice with a sub-sample of students (n=57), purposively selected based on socio-demographics and cyberbullying experience, twice with their parents (n=50), and once with their teachers (n=30).

**Results:**

Data collection for this study is complete. Planned analyses include transition probabilities and repeated measures analyses to determine involvement in cyberbullying. Repeated measures analyses, including between-subject factors (eg, socio-demographics), will be utilized to determine factors that protect or increase risk of involvement in cyberbullying. Qualitative analysis utilizing grounded theory is planned, to permit rich understanding of participant experiences and perspectives. Results will be reported in 2016 and 2017.

**Conclusions:**

This study will offer insight into the contemporary phenomenon of cyberbullying while also informing interventions to curb cyberbullying and address its pervasive social, mental health, and health consequences. Knowledge mobilization strategies and implications for research and practice are discussed.

## Introduction

### Information and Communication Technology Use Among North American Youth

Information and communication technologies (ICTs) are pervasive among socio-demographically diverse populations of young people in North America. Use of these technologies is increasingly mobile (eg, cell phones, smartphones, tablets). In the United States in 2014, 92% of adolescents (13-17 years) were online daily (56% several times per day), while 91% of youth went online occasionally, at minimum, through a mobile device [1,2]. The recent advances in ICTs offer immense benefits for children and youth, including innumerable and unprecedented opportunities for education, growth, and development [3-7], as well as facilitating their health and mental health [8]. The ever-growing ubiquity of ICTs has, however, inevitably brought new challenges [9,10]. Despite their technical proficiency, children and youth do not typically possess the critical thinking and decision-making abilities required to use technology safely [11], and may be exposed to significant risks in ICT environments, including cyberbullying.

### Cyberbullying: A Growing Public Health Problem

In the past few years, there has been an explosion in research on cyberbullying, documenting it as a serious, prevalent, and growing problem. Prevalence rates for cyberbullying vary due to definitional inconsistencies, the population studied, and the time frames and methodologies used [12,13,14]. It has been established, however, that between 10-40% of youth report being cyberbullied, while 50% know someone who has experienced cyberbullying [15]. Bullying is generally defined as a form of aggression that can be direct or indirect, and includes hostile physical, verbal, psychological, or relational behaviors. Bullying is characteristically intentional, commonly occurring in the context of a relationship, and comprising a power imbalance among those involved. The aggressive behavior is typically repeated over time, resulting in harm or negative consequences for the victimized child or youth [2]. Although consensus on the definition of cyberbullying has been difficult to establish, it may be generally defined as the use of ICTs to bully another person [15-21]. Young people may be involved in cyberbullying as victim, perpetrator, and/or witness. These roles appear to be more fluid and difficult to distinguish in the case of cyberbullying compared to traditional offline bullying [22]. Occurrence of bullying and cyberbullying are also highly correlated [14]. Research suggests that regardless of the role played in cyberbullying incidents, all children and youth can experience serious negative social, mental health, and health consequences as a result of involvement [19-25].

Cyberbullying constitutes a mounting public health problem, as both victimized youth and perpetrators may experience significant and prolonged distress [14,17], as well as an array of mental health concerns and problem behaviors. Victimized children and youth are at risk of developing depression, anxiety, poor self-esteem, eating disorders, sleep difficulties, emotional problems (eg, fear, sadness, loneliness), psychosomatic problems (eg, abdominal pains, headaches), and suicidal ideation and behavior [26-28]. Victimized youth may also be at increased risk of using substances, experiencing difficulties in school, participating in delinquent behavior, and engaging in unsafe sexual practices [29-31]. Youth who are perpetrators similarly experience increased risk of problems including depressive symptoms, substance use, aggression, and suicidal ideation, and may demonstrate less empathy and more conduct problems [23,32,33]. Students who are marginalized due to particular social markers (such as race/ethnicity, gender, religion, appearance, sexual orientation, socioeconomic status, or disability) may be disproportionately vulnerable to experiencing cyberbullying and associated negative social, mental health, and health consequences [34,35].

While research to date has illuminated a great deal about the nature and consequences of cyberbullying, several areas require further examination. Additionally, few studies have employed a longitudinal study design to assess trends in cyberbullying over time. The purpose of this paper is to describe the protocols implemented in a longitudinal and multi-perspective mixed-methods cohort study that contributes to the existing research by investigating several of these underdeveloped areas.

### Study Objectives

This study had five objectives: (1) to explore children/youth’s experiences, and children/youth’s, their parents’, and their teachers’ conceptions, definitions, and understanding of cyberbullying; (2) to explore how children/youth view the underlying motivations for cyberbullying; (3) to document the shifting prevalence rates of cyberbullying victimization, witnessing, and perpetration; (4) to identify risk and protective factors for cyberbullying involvement; and (5) to explore social, mental health, and health consequences of cyberbullying among children/youth. In this paper, the methods of the study are clearly outlined, and future quantitative and qualitative data analysis plans are discussed.

## Methods

### Sample

Three participant groups were included in the baseline study sample: (1) students in 4^th^ (n=160), 7^th^ (n=243), and 10^th^ (n=267) grades; (2) their teachers (n=103); and (3) their parents (n=246). A stratified random sampling strategy was utilized to select participants. First, a random sample of 19 schools was drawn from one of the largest school boards in North America [36], situated in Toronto, Canada, which is a large metropolitan city. Schools were stratified into three categories of need (low, medium, and high) based on an index developed by the school board that ranked schools on external challenges to student achievement. The school board developed this index using census data associated with the postal code of students attending each school. Neighborhood-level census data used to develop the index included income and education levels, ratio of households receiving social assistance, and ratio of single parent families [37]. The stratification of the sample based on this index ensured representation of ethno-cultural and socioeconomic diversity - factors that potentially impact access to ICTs, experiences of cyberbullying, and the manifestation of negative outcomes [1,38,39]. In year three of the study, 10 additional schools were recruited for participation in order to follow those students transitioning from elementary/middle school to middle/secondary school. A total of 29 schools participated in the study. All students in the selected grades at the original participating schools were offered the opportunity to participate, as were their parents and teachers.

**Figure 1:**
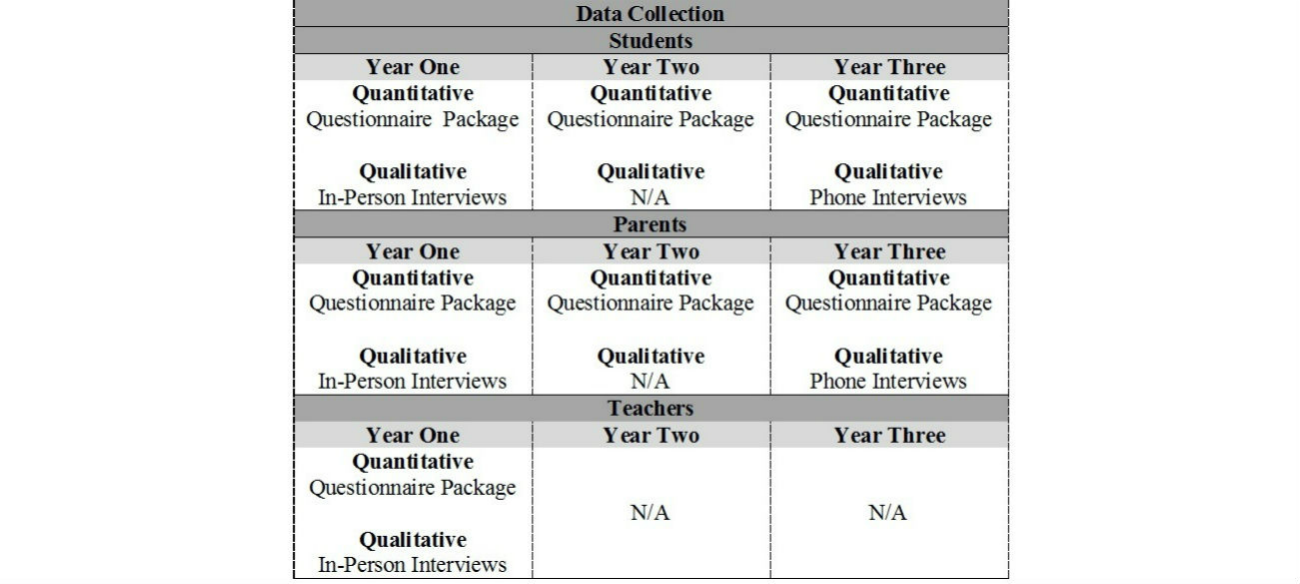
Study timeline.

Participating students and their parents provided data in all three years of the study, while matching teachers provided data in year one only (as student participants’ teachers changed each year). All three participant groups completed quantitative questionnaire packages, and a sub-sample of each group participated in qualitative interviews. Quantitative data were collected from students and parents in each year of the study, while qualitative data were collected only during years one and three, in order to allow for enough time to elapse for any changes in beliefs, perceptions, attitudes, and understanding of cyberbullying to emerge (Figure 1). Sub-samples of students, parents, and teachers were purposefully selected to participate in interviews based on level of school need, and were representative of gender, grade, and bullying/cyberbullying involvement.

### Study Team and Training of Research Assistants

The research team consisted of a principal investigator and five co-investigators (responsible for general study oversight), one research manager responsible for data management (including entry and cleaning), and four research coordinators. The research coordinators worked in collaboration, but were responsible for separate aspects of the project: (1) survey administration and overall coordination, (2) consent and maintenance of administrative databases, (3) qualitative interview coordination, and (4) coordination of supports for students identified as experiencing distress. Coordinators managed a team of approximately 10-15 research assistants (RAs) who held diverse and often multiple roles, including: collecting survey data in the school setting, collecting interview data in the school setting or by phone, following up with students in distress, and assisting with administrative tasks. Most RAs were in progress towards (or held) a Master of Social Work degree, while several were from other related professional faculties, such as public health and education.

Prior to working on the project, all RAs participated in a two-hour general training on study methods and procedures. RAs were then trained for specific roles and duties depending on their educational background, clinical experience, and interests. Training was provided for administering quantitative surveys in the school setting, conducting qualitative interviews in person or on the phone, completing assessments to evaluate whether a student was in distress, and accomplishing various administrative study tasks.

### Ethics and Consent Process

Ethics approval was sought and received from the University of Toronto Research Ethics Board (Protocol #26753). The External Research Review Committee at the partnering school board also provided ethics approval for this project.

Consent to participate in the study was obtained actively in year one and, with approval of the school board, passively in years two and three. In year one, RAs visited each 4^th^, 7^th^, and 10^th^ grade classroom from the 19 selected schools to explain the study and distribute consent forms. Parents/guardians were asked to sign the form if they agreed to allow their child to participate, if they were interested in participating themselves, and/or if they permitted the research team to ask their child’s teacher to participate. After collection of the parent/student consent forms, teachers were asked if they would like to participate, and completed a consent form. In years two and three (passive consent), parents/guardians were mailed a letter reminding them that they had consented for their child and/or themselves to participate in the study, and that the next year of the study was ready to commence. The letter also provided detailed instructions on how to withdraw from the study if desired.

A $5 gift card was offered to all students, teachers, and parents who participated in the quantitative survey portion of the research in each of the three years. A $10 gift card was offered to all participants who took part in an interview (in person or by telephone) in years one and three.

In anticipation that some questions could lead to distress or disclosure of information of a potentially sensitive or distressing nature, a Research Ethics Board-approved protocol (agreed upon by both the University and school board) was established to identify and assist students categorized as being *in distress* through their questionnaire and/or qualitative interview responses. Student participants were classified as *in distress* if they met one (or more) of the following five criteria: (1) indicated on the Bullying & Cyberbullying: Perpetrators, Victims & Witnesses Survey (B&C:PVWS) that they needed help and would like to speak to a researcher; (2) endorsed item related to fire-setting on the Youth Self Report (YSR); (3) endorsed items related to self-harm/suicide on the YSR; (4) scored above the 85th percentile on the YSR, which is indicative of experiencing numerous behavioral problems; and/or (5) indicated during qualitative interviews that they were highly stressed and in need of support. All children and youth identified as *in distress* were individually interviewed in a private and confidential school setting by a clinically trained researcher, who was a Master of Social Work student or who possessed equivalent education and experience [40]. Children and youth were interviewed regardless of whether the nature of their distress was bullying related. Participants were then connected to appropriate services established within the school board. This attention to the distress of participants was particularly salient in the research context, as access to mental health services in Canada remains problematic [41].

### Data Collection

#### Quantitative Data Collection Methods

In year one, students in 4^th^grade (n=160), 7^th^ grade (n=243), and 10^th^ grade (n=267) completed a 45-60 minute survey in the school setting, while parents (n=246) completed a 30-45 minute survey by mail. This procedure changed somewhat for years two and three of the study, with some students completing questionnaire packages by mail due to changing schools. Questionnaires for teachers (n=103), which took approximately 45-60 minutes to complete, were administered in the participating schools. Teachers were given approximately two weeks to complete the questionnaires about their students participating in the study, which were then collected by the research team.

#### Quantitative Data Collection Measures

This study utilized a variety of quantitative measures, including both standardized measures as well as measures developed specifically for the study (Table 1). Student, parent, and teacher surveys collected information regarding experiences with bullying/cyberbullying, socio-demographics, ICT use, and student mental health, health, social, and behavioral issues.

**Table 1:** Measures completed by students, parents, and teachers.

			Students			Parents	Teachers
			**Grade Level**				
**Area**	**Measure**	**Captures**	**4**	**7**	**10**		
Experiences with Bullying/ Cyberbullying	*Bullying & Cyberbullying: Perpetrators, Victims & Witnesses Survey*(B&C:PVWS)	Experiences as victims, perpetrators, and/or witnesses of both bullying/cyberbullying; Experiences with bullying/cyberbullying types (eg, physical, verbal, social, sexual); Experiences with (and content of) bullying/cyberbullying specific to a variety of socio-demographic factors (eg, race, sexual orientation, gender, disability, appearance, religion); Responses to bullying/cyberbullying; Thoughts about potential interventions to address bullying/cyberbullying. *Measure used to identify distress.*	✓	✓	✓		
Mental Health, Health, Social, & Behavioral Issues	*Youth Self-report*(YSR) [ 42]	Youth’s self-reported anxiety/depression, suicidal ideation, self-harm, somatic complaints, social, thought and attention problems, delinquent (eg setting fires) and aggressive (eg hurting others) behaviors; *Measure used to identify distress.*		✓	✓		
	*Child Behavior Checklist*(CBCL) [ 43]	Parent counterpart to YSR.				✓	
	*Teacher Report Form*(TRF) [ 44]	Educator counterpart to YSR.					✓
	*Self-Perception Profile for Children*(SPPC) [45]	Self-esteem.	✓	✓			
	*Self-Perception Profile for Adolescents*(SPPA) [ 46]	Self-concept.			✓		
	*Social Support Scale for Children*[ 47]	Children’s perceived support and regard from parents, teachers, close friends, and classmates.	✓	✓			
	*Social Support Behaviors Scale*[ 48]	Youth’s perceived support from family members and peers with subscales: emotional, socializing, practical assistance, financial, advice/guidance.			✓		
Socio-Demographics	Developed for the purpose of this study (Multiple Versions)	Gender, age, country of birth, country of parents’ birth, main language spoken at home, race/ethnicity, sexual orientation, disability, family composition, grades, and other socio-demographic characteristics. Four versions: (1) 4^th^Grade; (2) 7^th^and 10^th^Grade; (3) Parents; (4) Teachers.	✓	✓	✓	✓	✓
Information & Communication Technology Use	Developed for the purpose of this study (Multiple Versions)	Access to ICTs at home, activities while using ICTs, frequency of activities, online friends and connections. Four versions: (1) 4^th^Grade; (2) 7^th^and 10^th^Grade; (3) Parents; (4) Teachers.	✓	✓	✓	✓	✓

Children/youth’s experiences with bullying and cyberbullying were measured using the B&C:PVWS, which is a compilation of survey questions developed from the research team’s previous studies. The bullying and cyberbullying literature was reviewed and feedback was sought from the participating school board in order to ensure age-appropriate language. Specific questions were adapted or removed based on the feedback from the school board (eg, questions regarding online sex). For the questions measuring experiences of being bullied and bullying others, the Cronbach alphas were .77 and .71, respectively, indicating good internal consistency.

Children/youth’s mental health, health, social, and behavioral issues were captured for 4^th^, 7^th^ and 10^th^ grade cohorts. We captured mental health, health, and behavioral issues using the YSR, intended for children aged eleven and older [42]. Parents completed the Child Behavior Checklist (CBCL), which is the parental counterpart to the students’ YSR [43], while teachers completed the Teacher Report Form (TRF), which is the educator counterpart to the YSR and CBCL [44]. These surveys are widely used measures with excellent reported test-retest reliability [42-44]. We captured children/youth’s social issues, including self-esteem, using subscales from the Self-Perception Profile for Children (SPPC) [45] and the Self-Perception Profile for Adolescents (SPPA) [46]. These scales have adequate internal consistency, and both measures have a stable factor structure [45,46]. We measured social support for students in the 4^th^ and 7^th^ grade cohorts using the Social Support Scale for Children, a 24-item instrument which assesses children’s perceived support and regard from parents, teachers, close friends, and classmates [47]. The internal consistencies of the four subscales range from .72 to .88 using Cronbach alphas [47]. Adolescents in the 10^th^ grade cohort completed the Social Support Behaviors Scale to assess perceived support from family members and peers (emotional, social, practical assistance, financial, and advice/guidance) [48]. Strong internal consistency (Cronbach alpha exceeding .85) has been reported for this scale, which includes several college samples [48,49].

Socio-demographics were collected using two versions of the student demographic questionnaire, capturing characteristics such as age, gender, and country of birth, which were developed by the research team with feedback from the school board (one for the 4^th^ grade cohort and one for 7^th^ and 10^th^ grade cohorts). The questionnaires included similar items for both age groups and were based on previous instruments administered by the school board, instruments developed by co-investigators for similar studies, and a review of the literature. The questionnaire for the older cohorts included items regarding sexuality, which were not included in the version for 4^th^ grade students. Similar questionnaires (two versions) were developed for parents and teachers.

Lastly, we collected data on ICT use, using two versions of the student ICT usage questionnaire (one for the 4^th^ grade cohort and one for 7^th^ and 10^th^ grade cohorts), developed by the research team. Again, both included similar questions, soliciting information on access to ICTs at home, activities while using ICTs, frequency of activities (6-point scale, ranging from *never to more than once a day*), and online friends and connections. The questionnaire for older cohorts included items related to taking and distributing intimate and/or sexual photos, which were not included in the 4^th^ grade version. These questionnaires were adapted from two previous studies. Parents and teachers also completed ICT usage questionnaires (two versions) similar to those filled out by students.

#### Qualitative Data Collection Methods

Student participants from 4^th^ grade (n=20), 7^th^ grade (n=21), and 10^th^ grade (n=16) in the qualitative sub-sample were purposefully selected from the larger quantitative sample for qualitative interviews based on diversity of gender, grade, school need level, and whether they reported bullying/cyberbullying victimization, perpetration, and/or witnessing. Subsequent to selecting student participants, their teachers (n=30), and their parents (n=50) were also invited to participate in in-depth interviews. Interviews lasted approximately one hour, ranging in length from thirty to ninety minutes. All year one interviews (with students, parents, and teachers) took place in the school setting, and utilized a semi-structured interview guide. Following preliminary analysis, this interview guide was expanded and refined for use in the year three follow-up phone interviews with the students and parents (Multimedia Appendix 1). Interviews provided nuance and context to the information obtained through the quantitative measures. Areas explored included views and understanding of cyberbullying and how it compares with traditional offline bullying, experiences of online aggression, and others’ attitudes and responses to the issue. Questions were guided by existing literature and the research team’s considerable experience. Parent and teacher interviews included questions about their awareness and understanding of cyberbullying, their child or student’s involvement in cyberbullying, links between cyber and traditional bullying, supports, and their responses to cyberbullying.

#### Data Management

All participants were assigned a unique code to maintain anonymity. Participants’ names do not appear anywhere in the quantitative survey packages or qualitative transcripts. Paper surveys were scanned using Cardiff Teleform software, and entered into a project-specific IBM SPSS Statistics 22 database. Entry and cleaning of quantitative data took place throughout the study, and all cases were cross-referenced by hand twice (during entry and after preliminary data sets were compiled) to ensure accuracy of entries. Qualitative data were transcribed verbatim, anonymized, and prepared for analysis. The same unique identifiers were used to identify the qualitative interviews and quantitative surveys, in order to facilitate matching these two data sources for individual participants.

## Results

Data collection for this study is complete. Results of the proposed analyses, outlined below, will be reported in 2016 and 2017.

### Proposed Quantitative Data Analyses

Descriptive analyses will be conducted to calculate frequencies for categorical variables, and means and standard deviations for continuous variables. We will summarize socio-demographic variables among participants in each grade level (4, 7, 10) and differences between grades will be assessed using Student t-tests for continuous variables, and χ2 analyses or Fisher’s exact tests for categorical variables. Items for each outcome scale (eg, Social Support Scale for Children) will be summed to calculate total or subscale scores for each measure. Reliability of scaled measures will be described using Cronbach alphas. Advanced statistical analyses are also planned. An example of a more advanced analysis that will be conducted is transition probabilities, which will determine involvement in cyberbullying, consistent with our objective of documenting the shifting prevalence rates of cyberbullying victimization, witnessing, and perpetration. To meet our objective of identifying factors that protect against (or increase risk of) involvement in cyberbullying, between-subject factors will be included in a repeated measures analysis. These factors include demographic variables, CBCL scales, self-esteem, and social support to determine their individual and combined contribution to cyberbullying experiences. Considering participants are clustered in classrooms, independence of the data cannot be assumed, and the data are dependent to some degree. Thus, classroom will be included as a dummy variable in the regressions. Multilevel analysis will be used to assess the contribution of school need level (low, high, and medium) on individual cyberbullying experience.

### Proposed Qualitative Data Analyses

Using the systematic procedures of a rigorous grounded theory inquiry, a theory about children/youth’s, parents’, and teachers’ conceptions of cyberbullying and underlying motivations will be generated. Using this approach, researchers concurrently collect, analyze, and theorize about data in a reciprocal process of constant comparison to inductively construct empirically corroborated, explanatory theories [50-53]. The iterative process permits the analytical and theoretical categories developed by previously collected data to inform, as well as refine and focus, subsequent collection of data [52,54,55]. This refining and focusing commenced during data collection for this study, particularly between the qualitative interview phases (years 1 and 3), and is ongoing. With future analyses, emergent themes among youth, parents, and teachers over time will continue to be identified, and children’s and adults’ views compared.

While the intent is to develop a theoretical model, grounded theory methods will simultaneously allow for further exploration of interpersonal processes and experiences in a process of reciprocal analysis. Line-by-line and open coding of transcripts were, and will continue to be, conducted to establish preliminary analytic focuses, and subsequently emerging categories will be built and expanded. Axial coding will promote connections both within and between categories and sub-categories, and facilitate synthesis and explanation [50,51,56]. Several measures have been employed to ensure trustworthiness and authenticity. The researchers’ prolonged engagement through many years of research and practice in this area will guide development of the grounded theory. Theory development will continue until saturation occurs. Reflexive journaling, bracketing, an audit trail, and dense descriptions will further ensure trustworthiness and transferability [50,51,54].

## Discussion

The study described in this paper provides one of the first assessments of the understanding and experiences of children and youth involved in cyberbullying as victims, perpetrators, and/or witnesses, and involved the investigation of their perceptions, as well as those of their parents and teachers. We followed a baseline sample of 4^th^ (n=160), 7^th^ (n=243), and 10^th^ (n=267) grade children/youth and their parents (n=246), for three years (2012-2014), along with collecting baseline data from their teachers (n=103). This study’s multi-perspective approach allows for triangulated analysis of cyberbullying issues, and the design was strengthened by tracking participants longitudinally, during a period in which ICT use has continued to expand rapidly [57]. Recruiting students across grades/ages/socio-economic status permits the comparison of experiences across diverse socio-demographic groups and allows for an examination of trends in primary, middle, and secondary schools. Data collection for this study is complete, with results of proposed analyses anticipated in 2016 and 2017.

This research will elucidate the complex dynamics of cyberbullying incidents and contributes to the growing body of literature on the rates of cyberbullying, as well as risk and protective factors of involvement. In addition, this study will explicate how children/youth understand cyberbullying and how they experience and judge the underlying motivations for involvement. This inquiry addresses the lack of research capturing children and youth’s experiences, feelings, and conceptions of cyberbullying, and uniquely examines the congruence or incongruence of children and youth’s views with those of significant adults in their lives. Identifying how children, youth, and adults conceptualize cyberbullying is critical to ensuring the understanding of its extent and impact, and developing effective prevention and intervention strategies [15]. Developing informed strategies relevant to contemporary young people’s lives and contexts is especially salient, as increasing recognition of the negative consequences of cyberbullying “has lead parents, educators, and policymakers to embrace intervention efforts, and there is now substantial educational and clinical interest in programs that help to mitigate… harmful outcomes” [58]. For emerging findings based upon study objectives, please refer to Multimedia Appendix 2 .

Knowledge translation and exchange activities will be a priority in order to translate study findings for study participants, educators, helping/healthcare professionals, and the broader community. Presentations will be made to the partner school board and a report will be provided to schools, participants, and community members. Any requests by individual schools for presentations will be accommodated by a member of the research team. Findings will be disseminated within the academic community through refereed journals and presentations at juried Canadian and international conferences. We will publish in relevant academic journals, and results will be disseminated to policy makers and practitioners, and presentations will be made to professional organizations and to the community.

Most importantly, these findings can inform interventions to curb cyberbullying among young people in an effort to prevent the negative social, mental health, and health consequences. In keeping with the preliminary findings of this study, previous research has indicated that most children and youth do not disclose their experience with cyberbullying to parents, and are even less likely to disclose cyberbullying experiences to school-based adults (eg, teachers, administrators) [12]. Such lack of disclosure indicates a critical need to provide prevention and intervention efforts in school settings as a way to promote disclosure [12]. Further, little evidence for best practices in intervention efforts exists [13]. The study described in this paper can inform intervention efforts by offering insight into student perceptions of what is helpful or not helpful when experiencing, perpetrating, and/or witnessing cyberbullying, as well as the contexts in which prevention and intervention efforts may be most effective (including via ICTs) [12]. Results of our quantitative data analysis exploring the social, mental health, and health consequences of cyberbullying can inform the development of resources at the school-level. Moreover, future papers focusing on the research process of this study may glean important insights into the challenges of conducting longitudinal studies with children and youth in a school-based setting (ie, participant retention), and potential strategies to mitigate these challenges (ie, the use of passive consent). Future research may also focus on mechanisms, beyond built-in research study protocols, to support students in distress.

The burgeoning body of literature on the phenomenon of cyberbullying is a relatively recent scholarly development, highlighting the crucial need to engage in discourse regarding this emerging field of research. This unique study offers insight into cyberbullying and provides a foundation for future research in this important and flourishing field. Importantly, as the frequency of ICT use is constantly growing, and with younger and younger children increasingly using ICTs, understanding the social, mental health, and health consequences of cyberbullying across grade levels may point to differing developmental impacts and inform targeted interventions.

## Appendix

Multimedia Appendix 1 Qualitative interview guide.

Multimedia Appendix 2 Preliminary results.

## Abbreviations

B&C: PVWS: Bullying & Cyberbullying: Perpetrators, Victims & Witnesses Survey
CBCL: Child Behavior Checklist
ICT: Information and communication technology
RA: Research assistant
SPPA: Self-Perception Profile for Adolescents
SPPC: Self-Perception Profile for Children
TRF: Teacher Report Form
YSR: Youth Self-Report
