# Supplementary material for: Prevalence, Motivations, and Social, Mental Health and Health Consequences of Cyberbullying Among School-Aged Children and Youth: Protocol of a Longitudinal and Multi-Perspective Mixed Method Study
Source: JMIR Res Protoc. 2016 May 24;5(2):e83. doi: 10.2196/resprot.5292 (PMC4897298; doi:10.2196/resprot.5292)
Supplement: Multimedia Appendix 1 [file resprot_v5i2e83_app1.pdf]

Multimedia Appendix 1. Qualitative Interview Guides

STUDENTS (YEAR 3 GUIDE)

Domain 1: Questions on cyber interactions

Example question: Can you tell me about how YOU use cyber technology -e.g., cell phones, emails, internet, social networking, games, YouTube?

> **Probe:** How often do you use it? What do you use it for e.g. homework, online games, music, chatting, etc.?

Domain 2: Questions on bullying and cyberbullying

Example question: Have you ever experienced cyberbullying? Can you tell me about times when that has happened? – for example have you ever seen it happen, has it happened to you, or have you done it to others? (It can be 1, 2 or all 3 of these). - ASK 1 AT A TIME

> **Probe**: Identify clearly whether the participant is talking about being a target, witness, or perpetrator (or a combination). Your aim is to get a full picture of why, how, when, and where this happened, and what might have helped. Ask if friends were involved i.e. doing or witnessing the cyberbullying – did it affect the friendship? If participants talk about seeing a 'bad' photo of someone online, ask them for specific details about the photo (e.g. what makes it bad, find out the details of the photo). Ask about how they felt about the incident, and whether their feelings have changed since it happened.

Domain 3: Motivations of cyberbullying

Example question: What do you think kids get cyberbullied about? **Probe:** possible prompts (to be used only as last resort): appearance, race, gender, (dis)ability, school work etc. Try to get specifics

Domain 4: Difference between cyberbullying and face to face bullying

Example question: Do you think that being cyberbullied is different from being bullied face to face? Probe: Why? How? Do you think one is more serious? Which one? Why? If not considered different – find out why they think that. Find out if one is easier to do or deal with.

Domain 5: Getting help

Example question: Who do you think is responsible for helping young people deal with cyberbullying?

Domain 6: Follow-up

Example question: Do you think that your understanding of cyberbullying has changed since we last talked? If yes, how?

PARENTS (YEAR 3 GUIDE)

Domain 1: Questions on cyber interactions

Example question: Do you think your knowledge about cyber technology [e.g. the Internet and cell phones] is similar to your child's knowledge?
Probes: What do you think about the difference between how much you know about cyber technology and how much your child knows?

Domain 2: Questions on bullying and cyberbullying

Example question: Were you bullied/cyberbullied, did you bully/cyberbully, or did you witness bullying/ cyberbullying as a child? If so, could you tell me about it? What kind of effect/impact do you think it had on you? Do you think this affects how you respond to bullying incidents in your child's life?

Domain 3: Questions about the child

Example question: Has your child ever spoken to you about cyberbullying? If yes, how did you respond? Have you ever spoken to your child about cyberbullying? If yes, how did they respond? If no, why not?

Domain 4: Motivations of cyberbullying

Example question: Why do you think children and teens cyberbully each other? **Probe:** What are the motivations? Power? Technology makes it easy?
If these motivations are not mentioned, ask "Do you think it has anything to do with feeling powerful or being in control? Do you think that technology makes it easy?

Domain 5: Difference between cyberbullying and face to face bullying

Example question: Do you think that being cyberbullied is different from being bullied face to face? How so? **Probe:** Why? How? Do you think one is more serious? Which one? Why? If not considered different – find out why they think that. Find out if one is easier to do or deal with.

Domain 6: Getting help

Example question: What do you think would help children and youth who are being cyberbullied AND those who cyberbully?

Domain 7: Follow-up

Example question: Has anything changed for your child in their experience of bullying or cyberbullying since we last talked?

TEACHERS (YEAR ONE GUIDE)

Domain 1: Starting questions

Example question: Are you aware of real life incidents of cyberbullying in the school/class?

Domain 2: Experience questions

Example question: Have you been cyberbullied? If yes, please tell me about it

Domain 3: Questions about students

Example question: Do you think you have enough resources to educate/make students aware of cyberbullying and how to deal with it when it happens? If yes, please explain.

Domain 4: Perspective questions

Example question: Why do you think some children and youth get cyberbullied, while others don't?

Domain 5: Technology and bullying questions

Example question: Do you have any rules for your students, about their use of cyber technology [e.g. internet, cell phones]? If yes – could you explain? Do students follow the rules? If no rules in place- why?

Domain 6: Closing questions

Example questions: What responsibilities do you think you and your school has with regards to cyberbullying?
