# Supplementary material for: Prevalence, Motivations, and Social, Mental Health and Health Consequences of Cyberbullying Among School-Aged Children and Youth: Protocol of a Longitudinal and Multi-Perspective Mixed Method Study
Source: JMIR Res Protoc. 2016 May 24;5(2):e83. doi: 10.2196/resprot.5292 (PMC4897298; doi:10.2196/resprot.5292)
Supplement: Multimedia Appendix 2 [file resprot_v5i2e83_app2.pdf]

Multimedia Appendix 2. Emerging Findings

Objective I: To explore children/youth's experiences, and children/youth's, parents, and teachers' conceptions, definitions and understanding of cyberbullying

- Most articulated a relatively comprehensive definition of cyberbullying, and listed the potential negative outcomes associated with involvement as victimized, perpetrator, and/or witness.
- However, young people in particular simultaneously struggled to identify whether situations they experienced constituted cyberbullying. Normalization of cyberbullying led to the perception of "joking" among friends or peers, even if the student was distressed by the behaviour.
- A gendered component emerged, whereby boys' behavior was conceptualized as "trash talk" (often in online gaming) and girls' behavior was conceptualized as "drama" (often over social media).

Objective II: To explore how children/youth view the underlying motivations for cyberbullying;

- Cyberbullying was viewed as a way for perpetrators to gain a sense of power and control.
- Participants highlighted the quickly shifting nature of roles in interactions, emphasizing the difficulty in naming the perpetrator and victimized child or youth in many situations.
- Reciprocal negative interactions, wherein victimized students reclaimed power by engaging in aggression toward others, were also discussed.

Objective III: To document the shifting prevalence rates of cyberbullying victimization, witnessing and perpetration

- The quantitative sample was robust (T1=670, T2=565, T3=451). Overall, 423 students (61.2% retention) completed all three time points.
- There was a statistically significant increase in students reporting victimization between T1 (n=53/670; 7.9%) and T3 (n=44/451; 9.9%) [$\chi2(1)=8.975$, $P < .01$] and witnessing between T1 (n=147/670; 22.0%) and T3 (n=111/451; 24.7%) [$\chi2(1)=25.161$, $P < .001$]. Despite the increasing prevalence of victimization and witnessing, few students reported being perpetrators at T1 (n=17/670; 2.5%) and T3 (n=9/451; 2.0%), with insufficient numbers to calculate bivariate statistics.

Objective IV: To identify risk and protective factors for cyberbullying involvement

- Numerous risk and protective factors for cyberbullying involvement exist which are quite complex, including accessing or not accessing adult support. We hypothesized that coping strategies such as talking to adults (e.g., parents, teachers, helping professionals) may protect youth from cyberbullying involvement and/or ameliorate the negative consequences of cyberbullying. Yet, our preliminary findings indicate that while most young people in our study indicated that telling an adult (e.g., parents, educators, and/or helping professionals) as their primary strategy for coping with stressful life events, few students had actually implemented this strategy or expected they would do so if they experienced cyberbullying in the future.

Objective V: To explore social, mental health, and health consequences of cyberbullying amongst children/youth

- Almost one fifth of all year one participants were identified as being in distress.
- Of those in distress, 32% endorsed suicidal ideation.
- Female students, students with a disability, and students identifying as non-heterosexual were more likely to be in distress compared to male students, students without disabilities, and students identifying as heterosexual [54].
